# Free Fatty Acids and Complex Lipids in Patients With Severe Obesity Treated With Roux‐En‐Y Gastric Bypass: Impact of Diabetes Status

**DOI:** 10.1155/bri/1805140

**Published:** 2025-12-08

**Authors:** Freja Eriksen, Sten Madsbad, Mogens Fenger, Elin R. Carlsson

**Affiliations:** ^1^ Department of Clinical Biochemistry, Nordsjællands Hospital, University of Copenhagen, Hillerød, Denmark, ku.dk; ^2^ Department of Clinical Biochemistry, Copenhagen University Hospital Hvidovre, Hvidovre, Denmark, hvidovrehospital.dk; ^3^ Department of Endocrinology, Copenhagen University Hospital Hvidovre, Hvidovre, Denmark, hvidovrehospital.dk

**Keywords:** free fatty acids, phosphatidylcholine, Roux-en-Y gastric bypass, triglyceride, Type 2 diabetes

## Abstract

This study investigates the relationship between eight free fatty acids (FFA), triglycerides (TAG), and phosphatidylcholines (PC) after Roux‐en‐Y gastric bypass (RYGB) to obtain insight into changes in lipid metabolism that might be causally related to postsurgery Type 2 diabetes remission. We measured the FFA, TAG, and PC before and up to 2 years after RYGB in three patient groups: one nondiabetes group (*n* = 149), one diabetes group with postoperative remission (*n* = 33), and one diabetes group with persistent diabetes after surgery (*n* = 20). Pre‐ and postoperative Pearson’s correlations and linear regression models were used to assess the relationship between concentrations of individual FFA, TAG, and PC. At all timepoints, FFA explained more of the variance in PC than in TAG. The level of omega‐3 fatty acids was more strongly correlated to TAG in the group with diabetes remission compared to the nondiabetes group. The low plasma concentration of the omega‐6 fatty acid dihomo‐γ‐linolenic acid (DGLA) showed a surprisingly high correlation to both TAG and PC in all three groups. Unexpectedly, in the diabetes group without remission, a nonsignificant relation between saturated stearic acid and monounsaturated oleic acid indicates a possible impaired conversion of stearic acid to oleic acid via the enzyme delta‐9‐desaturase compared with the two other groups. We conclude that FFA might be involved in regulating the synthesis and metabolism of both TAG and especially PC. Diabetes status might influence the relationship between concentrations of FFA, TAG, and PC, and the data are suggestive of a role for the enzyme delta‐9‐desaturase in post‐RYGB diabetes remission.

## 1. Introduction

Metabolic and bariatric surgery, such as Roux‐en‐Y gastric bypass (RYGB), has become both an effective and safe treatment option for patients with obesity and diabetes [1]. Type 2 diabetes remission rates have been reported to be between 38% and 75% after RYGB surgery [1]. Glucose tolerance and insulin secretion improve even before substantial changes in body weight; thus, part of the mechanism behind postsurgery diabetes remission may be independent of weight loss [2]. This early improvement in glycemic control is thought to be caused in part by caloric restriction inducing significant increase in liver insulin sensitivity, and later, after major weight loss, an improvement is also seen in peripheral insulin sensitivity [3]. Postoperative anatomical and physiological alterations of the gastrointestinal tract resulting in enhanced secretion of several gut hormones might, however, also contribute to both the obtained weight loss and improved glucose homeostasis [4]. Thus, RYGB has been used as a unique model to investigate how the physiological changes seen postsurgery associate with diabetes remission, weight loss, and metabolism of complex lipids [5–7].

Several clinical models have been developed to predict the likelihood of diabetes remission after metabolic and bariatric surgery, including the DiaRem and ABCD. These models combine preoperative variables, such as age, diabetes duration, BMI, HbA1c, C‐peptide, insulin use, and number of antidiabetic medications to estimate the probability of remission [8]. Although they provide valuable clinical guidance, they do not address the underlying metabolic or lipidomic mechanisms, which remain poorly understood.

The status of lipid metabolism plays a major role in the pathophysiology of Type 2 diabetes [9]; in addition, dyslipidemia is closely and causally linked to the macrovascular complications in diabetes [10]. After RYGB, dyslipidemia is improved [11]. Typical lipid‐related biomarkers used to assess cardiometabolic risk in the daily clinic are serum concentrations of total cholesterol, high‐density lipoprotein (HDL) cholesterol, low‐density lipoprotein (LDL) cholesterol, and triglycerides (TAG) [12]. However, the role of the individual fatty acids as components of most lipids and the role of fatty acid composition in the determination of physiological, chemical, and physical properties of a lipid class need further elucidation.

HDL and LDL particles contain variable amounts of TAG [13], and TAG consists of three fatty acids and a glycerol backbone [14]. The concentration of saturated fatty acids, such as palmitic acid, has been shown to correlate with the risk of development of Type 2 diabetes, while, e.g., the omega‐6 fatty acid arachidonic acid and omega‐3 fatty acid levels have been shown to be inversely correlated to Type 2 diabetes [9]; therefore, the composition of fatty acids in TAG might be of special importance.

Phosphatidylcholines (PC), which is the most abundant phospholipid in mammalian cells [15], consist of two fatty acids and a choline group attached to a glycerol backbone [16] and are thus at least structurally closely related to TAG: Both are generated from diacylglycerols (DAG), but a third fatty acid is bound to DAG to form TAG [14], while instead a phosphocholine is coupled to DAG to generate PC [15]. PC are one of the main components in cell membranes.

A high content of saturated fatty acids in phospholipids is known to decrease fluidity of the cell membranes, which has been associated with decreased activity of the insulin receptor. In contrast, the presence of polyunsaturated fatty acids in plasma membrane phospholipids increases its fluidity and has been associated with improved insulin sensitivity [17].

Previously, we have measured and described changes after RYGB surgery of eight different free fatty acids (FFA) [7], PC [6], and TAG [5]. In this study, our aim was to investigate the following:

1. The association between serum concentrations of PC and TAG.
2. To what degree the changes in PC and TAG can be explained by changes in different plasma concentrations of FFA.
3. If the association between metabolically related FFA differs between diabetes groups. To our knowledge, no studies have previously investigated the relationship between changes in FFA, TAG, and PC after RYGB.

**Figure 1:**
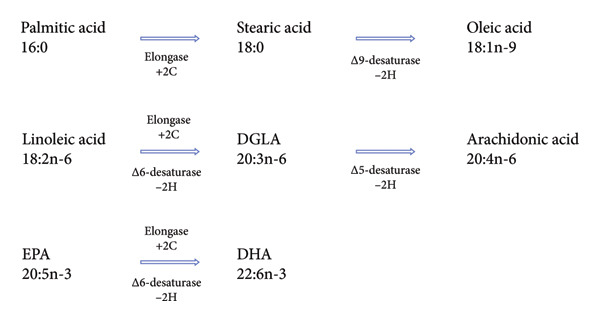
The eight measured FFA and their pathways. In the chemical formula, the first number indicates the amount of carbon atoms in the fatty acid carbon chain. The second number represents the amount of double bonds in the fatty acid. The number after the n refers to the location of the double bond closest to the methyl end of the fatty acid chain [32].

The eight FFA measured in this study belong to three distinct metabolic pathways (Figure 1): (1) the palmitic acid–stearic acid–oleic acid pathway, (2) the omega‐6 pathway, and (3) the omega‐3 pathway [18]. No cross‐metabolism occurs between these three pathways in mammals [19]. However, there are other pathways for palmitic acid, stearic acid, and oleic acid for which we have not measured the metabolites.

## 2. Material and Methods

The study population consists of 202 patients treated for obesity with RYGB surgery between November 2010 and September 2013 at the Copenhagen University Hospital Hvidovre in the Capital Region of Denmark and has been previously described [2, 5, 7].

In brief, the selected patients had delivered fasting plasma and serum blood samples both before surgery and up to 2 years after surgery amounting to 770 plasma samples and 764 serum samples. Samples were divided according to duration after surgery at 3, 6, 12, and 24 months after RYGB as described in Ref. [5, 7].

Samples were frozen at −80°C and stored between 6 months and 8 years at the time for the measurement of plasma FFA and serum PC [5–7]. TAG was measured on the day of sampling.

As previously described, the population was clustered into three groups according to diabetes status before and after surgery [5, 7]: a nondiabetes group (NDM, *n* = 149); a group of patients with Type 2 diabetes with postsurgery diabetes remission (DMH‐NDM, *n* = 33), and a group of patients with Type 2 diabetes with persistent diabetes after RYGB (DMH‐DMH, *n* = 20). At 24 months postsurgery, follow‐up samples were available for 80 of the 202 included patients (40%), comprising 60 without diabetes, 12 with diabetes in remission, and 8 with persistent diabetes.

Remission of Type 2 diabetes was defined as a stable decrease in glycated hemoglobin (HbA1c) to below 48 mmol/mol (6.5%) without antidiabetic medication.

Before RYGB, 17 patients (8% of the total cohort) were treated with insulin. The proportion of insulin users differed markedly between diabetes subgroups, with 18% in the remission group and 55% in the group with persistent diabetes, while none in the nondiabetic group received insulin therapy.

Postsurgery, patients were instructed to eat according to common practice after RYGB and to adhere to general international dietary recommendations [20, 21]. When weight was stabilized 1 year after surgery, the recommended daily calorie intake matched energy expenditure for the individual patient.

This study was performed in accordance with the Helsinki Declaration and was approved by the Scientific Ethics Committee of the Capital Region, Denmark, protocol number HD2009‐78, extended with the protocol number H‐6‐2014‐029, and by the Danish Data Protection Agency. Informed consent was obtained in writing from all the participants in this study.

### 2.1. Preparation and Analysis

We have previously described the methods for analysis of plasma FFA [7] and serum PC [6]. In brief, gas chromatography–mass spectrometry (GC‐MS) (Shimadzu, Japan) was used to quantify eight FFA: C16:0 (palmitic acid), C18:0 (stearic acid), C18:1n‐9 (oleic acid), C18:2n‐6 (linoleic acid), C20:3n‐6 (dihomo‐γ‐linolenic acid, DGLA), C20:4n‐6 (arachidonic acid), C20:5n‐3 (eicosapentaenoic acid, EPA), and C22:6n‐3 (docosahexaenoic acid, DHA) after removal of the esterified fatty acids. To isolate the nonesterified FFA, we added 100 μL of a premade mixture containing 5 μL of C17:0 internal standard (a fatty acid that is not produced in humans) and 0.4 M potassium hydroxide (KOH) in methanol to our plasma sample (100 μL). Afterward, hexane (1 mL) was added to separate the FFA from the EFAs. After isolating the FFA, we added 1 mL of 5% sulfuric acid in methanol to obtain the methylation needed to quantify the FFA with GC‐MS. The methylated FFA were dried under a stream of nitrogen and dissolved in 200 μL hexane after which the solution with fatty acid methyl esters was ready for injection and separation in the GC‐MS.

The fatty acid methyl ester derivatives of the FFA were separated on GC column, and the final identification of the FFA was performed using the MS fragment analysis conducted by the software library supplied by the vendor of the system, not from the retention times of the fatty acid methyl esters on the column.

To the Sigma‐Aldrich standards, we added methylated C17:0 in the amount needed to obtain similar concentrations as in the plasma samples. The standards with the C17:0 did not go through the same preparation as the plasma samples because both the standards and the internal standard were already methylated.

We used quadratic regression equations obtained from a five‐point calibration curve to determine concentrations of the 8 FFA. The curves showed high correlation coefficients (*r* > 0.996), indicating that the chosen regression is a good fit for our data.

The method for measurement of PC has previously been described [6]. In short, we measured serum PC levels by determining the phosphocholine content of PC with an in‐house enzymatic assay. A serum sample was incubated with a PC‐specific phospholipase, choline oxidase, and peroxidase. A standard solution of choline chloride was used to control assay levels. We used double determinations and analyzed all samples from individual patients at the same time. SpectraMax i3x (Molecular Devices) was used to determine endpoint absorbance.

TAG was analyzed on the fully automated Cobas 6000 (Roche Hitachi, Japan).

The methods for other routine measures, such as HbA1c, have been described previously [2].

## 3. Statistical Methods

Stepwise linear regression was used to determine to which degree the eight FFA can predict the level of PC and TAG, respectively. The FFA with a nonsignificant Pearson correlation to the dependent variable were excluded from the analysis to avoid overfitting. Baseline differences in clinical and biochemical parameters between groups were evaluated by one‐way ANOVA. To minimize potential confounding from these differences, all postsurgery changes were analyzed relative to each participant’s own presurgery value. Potential effects of statin treatment on lipid parameters were assessed where relevant. Homoscedasticity, normality, and linear relationship between the included FFA and the dependent variable were confirmed. Multicollinearity was suspected when the variance inflation factor was > 5.

We found normal distribution and homogeneity of variances of most parameters. One‐way ANOVA and Tukey’s post hoc test were used to compare presurgery clinical characteristics and ratios of PC and TAG between groups.

One‐way ANOVA was performed with a Welch–Satterthwaite correction (of degrees of freedom) followed by a Games–Howell post hoc test where Levene’s test of equality of variances was statistically significant.

For a few nonparametric parameters, differences between groups were found using the one‐way ANOVA. In these instances, significance was confirmed with a Kruskal–Wallis *H* test.

Where the highest *p* value was found with the Kruskal–Wallis *H* test, multiple Mann–Whitney *U* test was performed using the Bonferroni correction.

Homeostatic Model Assessment for Insulin Resistance (HOMA2‐IR) was calculated using fasting C‐peptide and fasting glucose.

Throughout the article, data are written as mean with a 95% confidence interval or standard deviation. Nominal *p*‐values lower than 0.05 were considered statistically significant. The IBM SPSS Version 25 was used for all analyses except the Fisher *r*‐to‐*z* transformation used to assess the significance of the difference between two correlation coefficients in groups with different numbers of participants where we used the calculator at http://vassarstats.net/rdiff.html.

## 4. Results

### 4.1. Presurgery Clinical Characteristics

Table 1 shows the preoperative clinical characteristics and biochemical parameters for the patients in the three groups. Notably, in the two groups of patients with diabetes, there was a higher age and lower total and LDL cholesterol levels compared to the group without diabetes. The difference in cholesterol could possibly be explained by a more frequent use of statin treatment in the diabetes groups. Compared to the group without diabetes, the diabetes group without remission had a lower BMI and numerically higher HOMA2‐IR, while the group obtaining diabetes remission had significantly higher HOMA2‐IR and lower HDL cholesterol and EPA. HOMA2‐IR did not differ between the two diabetes groups and was lower in the nondiabetic group. Linoleic acid was lower in the group with diabetes remission compared to both the nondiabetes group and the group with persistent diabetes. For gender and TAG, the ANOVA test was significant; however, no significant difference was found with post hoc testing between any of the three groups.

**Table 1:** Preoperative clinical characteristics and biochemical parameters for patients grouped according to diabetes status.

	NDM (*n* = 149) mean (SD)	DMH‐NDM (*n* = 33) mean (SD)	DMH‐DMH (*n* = 20) mean (SD)	Anova *p* value
Age (years)	42.0 (9.0)^b^	50.4 (8.2)^a^	51.5 (7.4)^a^	1 × 10^−8^
Gender (f/m)	111/38^a^	17/16^a^	9/11^a^	0.010
Height (cm)	171.1 (9.8)^a^	174.7 (8.0)^a^	171.2 (11.4)^a^	0.155
Weight (kg)	126.6 (22.3)^a^	127.5 (20.0)^a^	117.6 (19.7)^a^	0.203
BMI (kg/m^2^)	43.1 (5.9)^b^	41.7 (5.26)^ab^	40.0 (3.6)^a^	0.005
Systolic BP (mmHg)	126.9 (15.0)^a^	131.2 (12.8)^a^	128.2 (14.7)^a^	0.310
Diastolic BP (mmHg)	82.0 (11.1)^a^	81.5 (6.8)^a^	82.4 (10.1)^a^	0.958
HbA1_c_ (mmol/mol)	34.5 (3.8)^b^ (*n* = 142)	48.9 (11.9)^a^	55.2 (10.0)^a^	6 × 10^−8^
HOMA2‐IR	2.72 (1.1)^b^ (*n* = 140)	3.73 (1.5)^a^	3.97 (3.6)^ab^ (*n* = 17)	0.003
Total cholesterol (mmol/L)	5.00 (0.96)^b^ (*n* = 144)	4.25 (1.1)^a^	4.24 (1.2)^a^	8 × 10^−5^
HDL cholesterol (mmol/L)	1.20 (0.29)^b^ (*n* = 144)	1.05 (0.36)^a^	1.09 (0.35)^ab^ (*n* = 19)	0.028
LDL cholesterol (mmol/L)	3.11 (0.85)^b^ (*n* = 143)	2.38 (0.98)^a^ (*n* = 31)	2.27 (1.1)^a^ (*n* = 18)	3 × 10^−6^
VLDL cholesterol (mmol/L)	0.672 (0.30)^a^ (*n* = 143)	0.765 (0.30)^a^ (*n* = 31)	0.889 (0.47)^a^ (*n* = 18)	0.080
Triglycerides (mmol/L)	1.51 (0.74)^a^ (*n* = 145)	2.15 (2.0)^a^	2.11 (1.2)^a^	0.034
Statins (+/−)	20/129^b^	21/12^a^	15/5^a^	2 × 10^−8^
Phosphatidylcholines (mmol/L)	1.92 (0.37)^a^	1.88 (0.53)^a^	1.86 (0.44)^a^	0.692
Palmitic acid mmol/L	1.74 (0.46)^a^	1.82 (0.55)^a^	1.60 (0.46)^a^	0.267
Stearic acid mmol/L	0.531 (0.12)^a^	0.528 (0.13)^a^	0.496 (0.10)^a^	0.499
Oleic acid mmol/L	0.860 (0.23)^a^	0.933 (0.24)^a^	0.809 (0.32)^a^	0.141
Linoleic acid mmol/L	0.872 (0.25)^a^	0.870 (0.33)^a^	0.692 (0.25)^b^	0.016
DGLA mmol/L	0.0984 (0.040)^a^	0.0976 (0.050)^a^	0.0837 (0.028)^a^	0.318
Arachidonic acid mmol/L	0.462 (0.16)^a^	0.510 (0.20)^a^	0.454 (0.17)^a^	0.310
EPA mmol/L	0.0393 (0.022)^b^	0.0532 (0.030)^a^	0.048 (0.023)^ab^	0.025
DHA mmol/L	0.122 (0.047)^a^	0.140 (0.057)^a^	0.122 (0.056)^a^	0.164

*Note:* Values are the means (SD) of 149 (NDM), 33 (DMH‐NDM), or 20 (DMH‐DMH) patients. *p* value is from one‐way ANOVA comparing the three patient subgroup means. Values within a row with different letters are significantly different (*p* < 0.05). Post hoc *p* values from Tukey’s and Games–Howell were performed but are not shown in table. If the number of patients with available clinical data was less than 95% of the total patients in the group, the actual number is specified. HbA_1c_, glycated hemoglobin; DGLA, dihomo‐γ‐linolenic acid; NDM, patients without diabetes mellitus (DM); DMH‐NDM, patients with DM in remission after Roux‐en‐Y gastric bypass (RYGB) surgery; DMH‐DMH, patients with DM not in remission after RYGB.

Abbreviations: DHA, docosahexaenoic acid; EPA, eicosapentaenoic acid; HDL cholesterol, high‐density lipoprotein cholesterol; HOMA2‐IR, Homeostatic Model Assessment for Insulin Resistance; LDL cholesterol, low‐density lipoprotein cholesterol; SD, standard deviation; VLDL cholesterol, very‐low‐density lipoprotein cholesterol.

The differences between groups in LDL cholesterol and total cholesterol remained significant after controlling for the effect of statin treatment. However, the significant differences for linoleic acid and EPA were nonsignificant after controlling for statin treatment.

Height, body weight, systolic and diastolic blood pressure, PC, palmitic acid, stearic acid, oleic acid, DGLA, arachidonic acid, and DHA were similar in the three groups before surgery.

### 4.2. Linear Relationship Between Plasma FFA in the Three Groups Before Surgery

In the metabolic pathway–specific linear regressions shown in Table 2 and Figure 2, the slope coefficient was significantly different from zero in all three groups except for the group with persistent diabetes, where the slope coefficient in the regression for stearic acid and oleic acid was nonsignificant. This might, in part, be caused by the low number of participants in this group; however, the standardized slope coefficient was only 0.175 compared to 0.519 and 0.629 in the nondiabetes group and the remission group, respectively, indicating that the lack of significance reflects an impairment of the metabolic pathway between the saturated stearic and the monounsaturated oleic acid. Also, the highly significant pathway relation between DGLA and arachidonic acid in the nondiabetes and diabetes remission groups was only marginally significant in the group with persistent diabetes. In contrast, diabetes status does not seem to influence conversion of the omega‐3 fatty acids EPA to DHA.

**Table 2:** Linear regressions of FFA reflecting the metabolism in the respective three pathways presurgery.

Presurgery	NDM *N* = 149		DMH‐NDM *N* = 33		DMH‐DMH *N* = 20	
Independent and dependent variables	Standardized slope coefficient	*p* value	Standardized slope coefficient	*p* value	Standardized slope coefficient	*p* value
IV = Palmitic acid	0.669	1 × 10^−20^	0.751	5 × 10^−7^	0.507	0.022
DV = Stearic acid						

IV = Stearic acid	0.519	1 × 10^−11^	0.629	9 × 10^−5^	0.175	0.461
DV = Oleic acid						

IV = Linoleic acid	0.512	2 × 10^−11^	0.669	2 × 10^−5^	0.704	0.001
DV = DGLA						

IV = DGLA	0.729	5 × 10^−26^	0.581	4 × 10^−4^	0.489	0.029
DV = Arachidonic acid						

IV = EPA	0.641	1 × 10^−18^	0.671	2 × 10^−5^	0.836	5 × 10^−6^
DV = DHA						

*Note:* NDM, patients without diabetes mellitus; DMH‐NDM, patients with Type 2 diabetes in remission after Roux‐en‐Y gastric bypass (RYGB) surgery; DMH‐DMH, patients with Type 2 diabetes not in remission after RYGB.

Abbreviations: DV, dependent variable; IV, independent variable.

Figure 2Linear regression between plasma stearic acid and oleic acid concentrations in the three study groups before Roux‐en‐Y gastric bypass (RYGB) surgery. Scatter plots with regression lines are shown for: (a) nondiabetes group (NDM), (b) Type 2 diabetes group with postoperative remission (DMH‐NDM), and (c) Type 2 diabetes group without remission (DMH‐DMH). Stearic acid and oleic acid concentrations are expressed in mmol/L.

**Figure:**
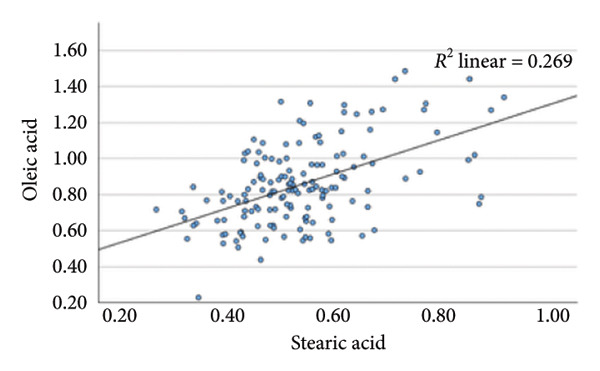
(a)

**Figure:**
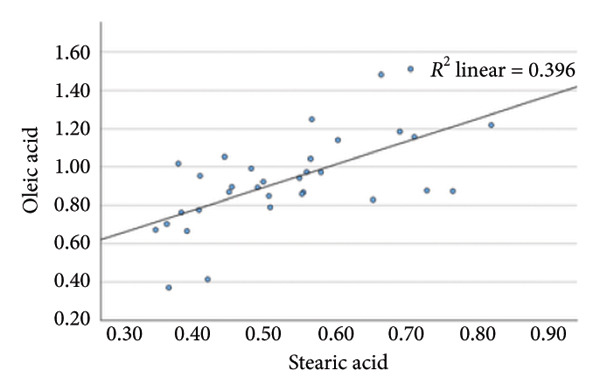
(b)

**Figure:**
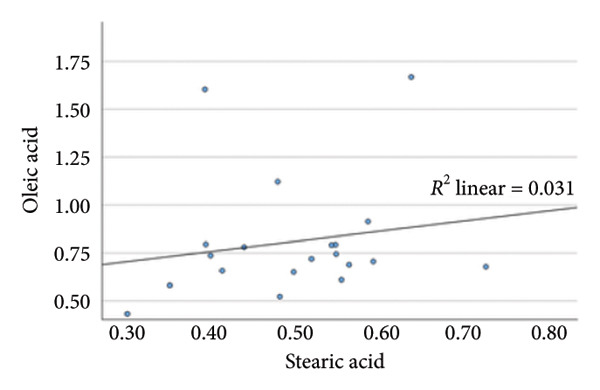
(c)

### 4.3. Linear Relationship Between PC and TAG Before and After Surgery

A significant correlation between PC and TAG was demonstrated presurgery in the nondiabetes group (*R* ^2^ = 0.401) and in the remission group (*R* ^2^ = 0.560), where correlations remained significant in the follow‐up period except at 6 months and 24 months postsurgery in the remission group (Table 3 and Figure 3). However, no significant correlation between PC and TAG was found for the group without diabetes remission at any timepoint except a marginally significant correlation at presurgery.

**Table 3:** Linear regressions of phosphatidylcholines on triglycerides before and after surgery.

Triglycerides	Slope coefficient (CI)	*p* value	Adjusted *R* ^2^
NDM			
Presurgery, *n* = 145	317.5 (253.3–381.4)	1 × 10^−17^	0.401
3 months, *n* = 149	313.8 (211.4–416.2)	1 × 10^−8^	0.200
6 months, *n* = 107	262.6 (116.2–409.0)	0.001	0.099
12 months N = 107	381.9 (221.3–542.4)	8 × 10^−6^	0.183
24 months, *n* = 60	364.4 (174.5–554.3)	3 × 10^−4^	0.203
DMH‐NDM			
Presurgery, *n* = 33	205.4 (140.6–270.2)	3 × 10^−7^	0.560
3 months, *n* = 33	317.0 (194.5–439.4)	1 × 10^−5^	0.457
6 months, *n* = 20	293.9 (−17.09–604.9)	0.063	0.134
12 months N = 16	287.5 (83.9–491.0)	0.009	0.353
24 months, *n* = 12	336.1 (−195.17–867.4)	0.189	0.082
DMH‐DMH			
Presurgery, *n* = 20	160.8 (3.097–318.6)	0.046	0.159
3 months, *n* = 20	252.1 (−132.7–636.9)	0.186	0.045
6 months, *n* = 12	241.3 (−341.9–824.5)	0.378	−0.014
12 months N = 15	186.7 (−171.3–544.8)	0.278	0.022
24 months, *n* = 8	78.15 (−401.2–557.5)	0.704	0.037

*Note:* Linear regression with phosphatidylcholines as the dependent variable and triglycerides as the independent variable before surgery and 3, 6, 12, and 24 months after RYGB in the three groups. NDM, patients without diabetes mellitus; DMH‐NDM, patients with Type 2 diabetes in remission after Roux‐en‐Y gastric bypass (RYGB) surgery; DMH‐DMH, patients with Type 2 diabetes not in remission after RYGB.

Abbreviation: CI, confidence interval.

Figure 3Linear regression between serum triglyceride (TAG) and phosphatidylcholine (PC) concentrations in the three study groups before Roux‐en‐Y gastric bypass (RYGB) surgery. Scatter plots with regression lines are shown for: (a) nondiabetes group (NDM), (b) Type 2 diabetes group with postoperative remission (DMH‐NDM), and (c) Type 2 diabetes group without remission (DMH‐DMH). PC and TAG concentrations are expressed in mmol/L.

**Figure:**
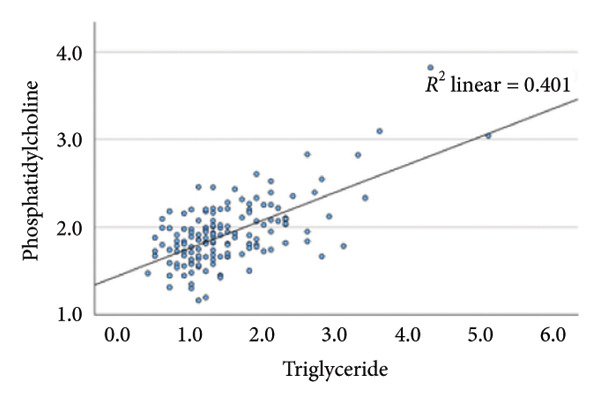
(a)

**Figure:**
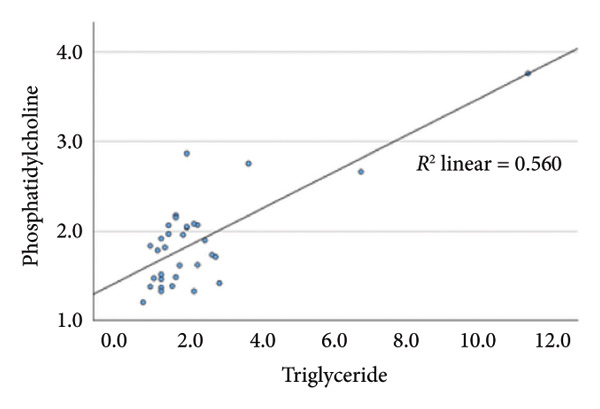
(b)

**Figure:**
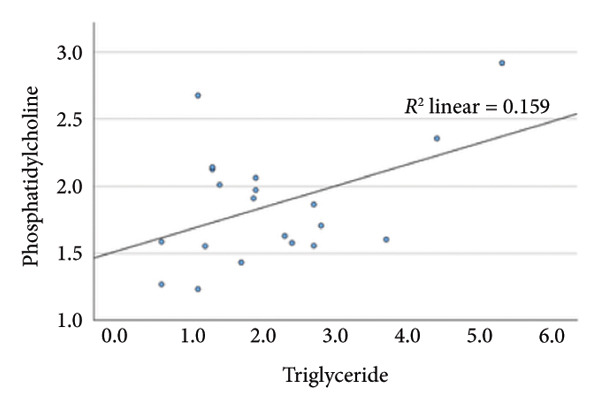
(c)

With a few exceptions (at presurgery and in the remission group at 3 months postsurgery), the ratio of PC and TAG between groups was not significant (Table 4).

**Table 4:** Comparing the phosphatidylcholines/triglycerides ratio in the three diabetes groups.

PC/TAG	NDM	DMH‐NDM	DMH‐DMH	*p* value
Molar ratio (mmol/L)/(mmol/L)	Mean ratio (95% CI) *N* = 148	Mean ratio (95% CI) *N* = 33	Mean ratio (95% CI) *N* = 20	
Presurgery (*N* = 145, 33, 20)act	1.391 (1.305–1.482)^b^	1.028 (0.884–1.195)^a^	1.004 (0.781–1.290)^a^	3 × 10^−5^
3 months (*N* = 149, 33, 20)	1.556 (1.486–1.630)^b^	1.362 (1.231–1.508)^a^	1.357 (1.508–1.601)^ab^	0.017
6 months (*N* = 107, 33, 20)	1.818 (1.722–1.919)^a^	1.555 (1.364–1.773)^a^	1.766 (1.683–1.853)^a^	0.070
12 months (*N* = 96, 16, 14)	2.048 (1.930–2.173)^a^	1.906 (1.558–2.333)^a^	1.729 (1.342–2.128)^a^	0.142
24 months (*N* = 60, 12, 8)	2.197 (2.014–2.396)^a^	1.882 (1.297–2.730)^a^	2.143 (1.590–2.887)^a^	0.690

*Note:* Values are the means (95% CI) of 148 (NDM), 33 (DMH‐NDM), or 20 (DMH‐DMH) patients. One‐way ANOVA (or Kruskal–Wallis H test) is comparing the three patient subgroup PC/TAG ratio means at either 3, 6, 12, and 24 months. Values within a row with different letters are significantly different (*p* < 0.05). Post hoc *p* values from Tukey’s and Games–Howell were performed but are not shown in table. Phosphatidylcholines (PC)/triglycerides (TAG) ratio values and ANOVA comparing the three diabetes groups. Data are reported as mean (95% CI). CI, confidence interval; NDM, patients without diabetes mellitus (DM); DMH‐NDM, patients with DM in remission after Roux‐en‐Y gastric bypass (RYGB) surgery; DMH‐DMH, patients with DM not in remission after RYGB.

### 4.4. Linear Relationship Between TAG and Individual Free Fatty Acids Before and After Surgery

Before surgery, in all groups, the four FFA with the highest Pearson correlation to TAG were palmitic acid, stearic acid, oleic acid, and DGLA. The correlations for these four FFA all tended to decrease until 6 months after surgery after which they again increased. The two omega‐3 fatty acids EPA and DHA showed a low correlation to TAG in the NDM group in general; however, they were significantly more strongly correlated to TAG at most of the timepoints in the diabetes group with remission (Table 5) (Fisher’s *r*‐to‐*z* test comparing the nondiabetes group with the group with remission. EPA: presurgery, *p* = 0.032; 3 months, *p* = 0.003; 6 months, *p* = 0.029; 12 months, *p* = 0.177; 24 months, *p* = 0.091 and DHA: presurgery, *p* = 0.030; 3 months, *p* = 0.021; 6 months, *p* = 0.834; 12 months, *p* = 0.030; 24 months, *p* = 0.289).

**Table 5:** Correlations between triglycerides and individual free fatty acids before and after surgery.

Triglycerides	Presurgery		3 months		6 months		12 months		24 months	
	P. corr.	VIF^∗^	P. corr.	VIF^∗^	P. corr.	VIF^∗^	P. corr.	VIF^∗^	P. corr.	VIF^∗^
NDM	*N* = 144		*N* = 149		*N* = 107		*N* = 98		*N* = 65	
Palmitic acid	**0.463**	2.75	**0.417**	2.71	**0.256**	2.57	**0.405**	1.88	**0.439**	2.77
Stearic acid	**0.452**	1.90	**0.315**	1.60	**0.124**	1.97	**0.331**	1.71	**0.410**	1.75
Oleic acid	**0.394**	2.97	**0.433**	2.82	**0.390**	2.60	**0.326**	1.80	**0.439**	3.27
Linoleic acid	**0.204**	2.29	**0.320**	2.85	**0.188**	2.60	0.069		0.119	
DGLA	**0.501**	2.37	**0.382**	1.93	**0.283**	2.30	**0.475**	1.62	**0.617**	2.41
Arachidonic acid	**0.319**	2.81	**0.278**	2.53	**0.197**	2.33	**0.278**	1.69	**0.242**	2.48
EPA	−0.007		−0.013		0.024		0.079		0.055	
DHA	0.128		**0.189**	1.68	**0.248**	1.42	0.117		0.081	
DMH‐NDM	*N* = 33		*N* = 33		*N* = 21		*N* = 16		*N* = 12	
Palmitic acid	**0.515**	3.67	**0.609**	6.13	0.361		0.345		0.460	
Stearic acid	**0.595**	2.56	**0.628**	4.82	**0.434**	1.62	**0.483**	2.39	0.365	
Oleic acid	**0.500**	2.44	**0.714**	3.79	0.315		**0.713**	5.93	0.327	
Linoleic acid	0.218		**0.368**	2.38	0.098		0.277		−0.072	
DGLA	**0.463**	2.82	**0.547**	2.37	**0.499**	1.97	**0.480**	1.49	**0.504**	1.26
Arachidonic acid	0.244		**0.399**	2.35	−0.117		**0.496**	5.66	0.172	
EPA	**0.400**	1.99	**0.530**	3.35	**0.523**	1.87	**0.446**	3.95	**0.576**	1.26
DHA	**0.511**	2.92	**0.575**	5.74	0.198		**0.640**	6.44	0.429	
DMH‐DMH	*N* = 20		*N* = 20		*N* = 13		*N* = 14		*N* = 10	
Palmitic acid	**0.667**	3.29	**0.571**	1.13	**0.632**	3.73	**0.485**	2.30	0.304	
Stearic acid	**0.409**	1.59	0.310		0.179		0.253		−0.071	
Oleic acid	**0.557**	4.58	**0.572**	1.13	**0.758**	1.98	**0.745**	2.90	0.372	
Linoleic acid	0.373		0.216		0.172		0.233		0.233	
DGLA	**0.582**	1.51	0.295		**0.601**	3.85	**0.464**	1.91	**0.762**	1.00
Arachidonic acid	**0.244**	2.86	0.196		**0.533**	1.53	**0.648**	1.97	0.400	
EPA	0.113		−0.196		−0.051		0.168		−0.438	
DHA	0.226		0.076		0.388		**0.466**	2.07	−0.253	

*Note:* Pearson correlation between log10 (triglycerides) and the 8 free fatty acids (palmitic acid, stearic acid, oleic acid, DGLA, arachidonic acid, EPA, and DHA) in the three groups plus. DGLA, dihomo‐γ‐linolenic acid; NDM, patients without diabetes mellitus; DMH‐NDM, patients with Type 2 diabetes in remission after Roux‐en‐Y gastric bypass (RYGB) surgery; DMH‐DMH, patients with Type 2 diabetes not in remission after RYGB. The correlations written in bold had a significant (< 0.05) Pearson correlation with log10 (triglycerides) and were therefore included in the stepwise multiple regression model.

Abbreviations: DHA, docosahexaenoic acid; EPA, eicosapentaenoic acid; P. Corr, Pearson’s correlation; VIF, variance inflation factor.

^∗^Variance inflation factor of the free fatty acids in the final linear regression model (after excluding the free fatty acids with insignificant Pearson’s correlation).

The FFA significantly correlated to TAG (written in bold in Table 5) were incorporated in the linear regression analysis for the respective timepoints and groups. In the nondiabetes group, the adjusted *R* ^2^‐values of the linear regression models at the respective timepoints ranged from 0.198 to 0.407, whereas they ranged from 0.235 to 0.494 and 0.427 to 0.544 in the diabetes groups with and without remission, respectively (Table 6). Thus, the FFA explained more of the variance in TAG in the two diabetes groups compared to the nondiabetes group, and in the group without remission, the FFA explained as much as about half of the variance in TAG at all timepoints. In the nondiabetes group, DGLA dominated compared to the other FFA with the strongest correlation to TAG except at 3 and 6 months postsurgery where oleic acid had the strongest correlation (Table 6). The stronger correlation between the two omega‐3 fatty acids EPA and DHA in the remission group seen in Table 5 was reflected in Table 6 where EPA explained the TAG variance without any other FFA contributing significantly to the regression at 6 and 24 months postsurgery.

**Table 6:** Stepwise linear regression for triglycerides and free fatty acids.

Triglycerides	Standardized slope coefficient	*p* value	Adjusted *R* ^2^	*F*‐change	*F*‐change *p* value
**NDM**					
Presurgery, *n* = 144					
**DGLA**	**0.378**	**5 × 10** ^**−**^ **^6^**	**0.254**	**49.7**	**7 × 10** ^**−**^ **^11^**
**Stearic acid**	**0.275**	**0.001**	**0.308**	**12.1**	**0.001**
Palmitic acid			0.320	3.43	0.066
3 months, *n* = 149					
**Oleic acid**	**0.371**	**3 × 10** ^**6**^	**0.182**	**34.0**	**3 × 10** ^**8**^
**Stearic acid**	**0.199**	**0.011**	**0.213**	**6.69**	**0.011**
EPA			0.222	2.809	0.096
6 months, *n* = 107					
**Oleic acid**	**0.453**	**9 × 10** ^**−**^ **^7^**	**0.198**	**27.139**	**9 × 10** ^**−**^ **^7^**
Palmitic acid			0.211	2.815	0.096
12 months, *n* = 98					
**DGLA**	**0.356**	**4 × 10** ^**−**^ **^3^**	**0.217**	**27.9**	**8 × 10** ^**−**^ **^7^**
**Palmitic acid**	**0.273**	**0.006**	**0.271**	**8.05**	**0.006**
Arachidonic acid			0.267	0.586	0.446
24 months, *n* = 65					
**DGLA**	**0.801**	**4 × 10** ^**−**^ **^8^**	**0.370**	**38.7**	**5 × 10** ^**8**^
**Arachidonic acid**	**−0.281**	**0.031**	**0.407**	**4.89**	**0.031**
Palmitic acid			0.385	0.033	0.856
**DMH-NDM**					
Presurgery, *n* = 33					
**Stearic acid**	**0.595**	**3 × 10** ^**−**^ **^4^**	**0.333**	**17.0**	**3 × 10** ^**−**^ **^4^**
DHA			0.350	1.846	0.184
3 months, *n* = 33					
**Oleic acid**	**0.714**	**3 × 10** ^**−**^ **^6^**	**0.494**	**32.3**	**3 × 10** ^**−**^ **^6^**
DGLA			0.524	2.89	0.099
6 months, *n* = 21					
**EPA**	**0.523**	**0.015**	**0.235**	**7.16**	**0.015**
DGLA			0.242	1.167	0.294
12 months, *n* = 16					
**Oleic acid**	**0.713**	**0.002**	**0.473**	**14.4**	**0.002**
DHA			0.482	1.24	0.286
24 months, *n* = 12					
**EPA**	**0.576**	**0.050**	**0.265**	**4.96**	**0.05**
DGLA			0.273	1.11	0.319
**DMH-DMH**					
Presurgery, *n* = 20					
**Arachidonic acid**	**0.449**	**0.030**	**0.446**	**16.3**	**0.001**
**Palmitic acid**	**0.418**	**0.041**	**0.544**	**4.87**	**0.041**
Oleic acid			0.570	2.011	0.175
3 months, *n* = 20					
**Oleic acid**	**0.427**	**0.033**	**0.290**	**8.74**	**0.008**
**Palmitic acid**	**0.426**	**0.034**	**0.427**	**5.32**	**0.034**
6 months, *n* = 13					
**Oleic acid**	**0.758**	**0.003**	**0.536**	**14.869**	**0.003**
Arachidonic acid			0.535	0.970	0.348
12 months, *n* = 14					
**Oleic acid**	**0.745**	**0.002**	**0.518**	**15.0**	**0.002**
Arachidonic acid			0.552	1.94	0.192
24 months, *n* = 10					
**DGLA**	**0.762**	**0.010**	**0.528**	**11.1**	**0.010**

*Note:* Stepwise linear regression with triglycerides as the dependent variable and the eight free fatty acids (palmitic acid, stearic acid, oleic acid, DGLA, arachidonic acid, EPA, and DHA) as the independent variables (as triglycerides have been log10 transformed to achieve the assumption of normality, the slope coefficient is the increase of triglycerides in percent per unit increase in the independent variable). DGLA, dihomo‐γ‐linolenic acid; NDM, patients without diabetes mellitus; DMH‐NDM, patients with Type 2 diabetes in remission after Roux‐en‐Y gastric bypass (RYGB) surgery; DMH‐DMH, patients with Type 2 diabetes not in remission after RYGB. The slope coefficient, standardized slope coefficient, *p* value, and variance inflation factor are from the final model including only the free fatty acids with significant contribution to the model. Adjusted *R* ^2^, *F*‐change, and *F*‐change *p* value reflect the stepwise addition of the respective free fatty acids in the model. The steps marked with bold are the FFA included in the model, while the steps not marked with bold are showing the adjusted *R*‐value and *F*‐change for the adding of the first FFA with an insignificant contribution to the model which is therefore not a part of the final model. The variance inflation factor was < 5 for all regressions.

Abbreviations: DHA, docosahexaenoic acid; EPA, eicosapentaenoic acid.

### 4.5. Linear Relationship Between PC and Individual Free Fatty Acids Before and After Surgery

**Figure 4:**
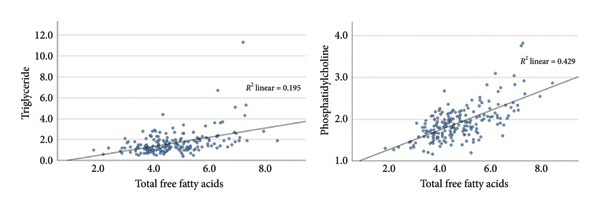
Linear regression between total plasma free fatty acids (FFA) and (a) serum triglycerides (TAG) and (b) serum phosphatidylcholines (PC) in the combined study population before Roux‐en‐Y gastric bypass (RYGB) surgery. Total FFA represents the sum of the eight measured free fatty acids (palmitic acid, stearic acid, oleic acid, linoleic acid, dihomo‐γ‐linolenic acid, arachidonic acid, eicosapentaenoic acid, and docosahexaenoic acid). All concentrations are expressed in mmol/L.

Compared to the correlations between FFA and TAG, the correlations between FFA and PC were markedly stronger (Table 7 and Figure 4). Presurgery, DGLA had the strongest correlation to PC of all FFA in all three groups except in the diabetes group without remission where it had the second strongest correlation next to stearic acid (Table 7).

**Table 7:** Correlations between phosphatidylcholines and individual free fatty acids before and after surgery.

Phosphatidylcholines	Presurgery		3 months		6 months		12 months		24 months	
	P. corr.	VIF^∗^	P. corr.	VIF^∗^	P. corr.	VIF^∗^	P. corr.	VIF^∗^	P. corr.	VIF^∗^
NDM	*N* = 148		*N* = 148		*N* = 110		*N* = 98		*N* = 59	
Palmitic acid	**0.556**	2.86	**0.597**	2.72	**0.586**	2.58	**0.451**	1.98	**0.666**	2.64
Stearic acid	**0.608**	2.10	**0.521**	1.61	**0.565**	2.05	**0.501**	1.80	**0.671**	1.59
Oleic acid	**0.537**	3.03	**0.410**	2.81	**0.473**	2.65	**0.305**	2.23	**0.454**	3.33
Linoleic acid	**0.357**	2.34	**0.457**	2.85	**0.572**	2.66	0.295	1.92	**0.426**	2.58
DGLA	**0.671**	2.50	**0.455**	1.94	**0.567**	2.18	**0.518**	1.16	**0.535**	2.18
Arachidonic acid	**0.522**	3.45	**0.440**	2.66	**0.471**	2.42	**0.274**	2.23	**0.385**	2.77
EPA	**0.312**	1.86	**0.224**	2.25	**0.381**	2.39	0.186	1.72	**0.169**	1.30
DHA	**0.270**	2.39	**0.402**	3.33	**0.471**	2.76	0.247	2.21	0.093	
DMH‐NDM	*N* = 33		*N* = 33		*N* = 20		*N* = 18		*N* = 13	
Palmitic acid	**0.752**	3.92	**0.635**	6.13	**0.608**	3.55	**0.476**	1.96	**0.685**	16.2
Stearic acid	**0.694**	2.60	**0.586**	4.82	**0.597**	1.63	**0.596**	2.39	**0.814**	15.1
Oleic acid	**0.688**	3.32	**0.659**	3.79	**0.384**	4.40	**0.671**	4.57	**0.653**	17.6
Linoleic acid	**0.598**	2.97	**0.517**	2.38	**0.498**	4.90	0.308		**0.755**	5.28
DGLA	**0.789**	2.87	**0.621**	2.37	**0.706**	1.98	**0.457**	1.40	**0.688**	3.15
Arachidonic acid	**0.571**	2.21	**0.533**	2.58	**0.528**	3.60	**0.722**	3.70	**0.859**	100.1
EPA	**0.305**	2.42	**0.539**	3.35	**0.517**	2.82	0.326		**0.859**	13.2
DHA	**0.611**	3.59	**0.666**	5.74	**0.576**	5.97	**0.582**	2.18	**0.870**	100.2
DMH‐DMH	*N* = 20		*N* = 20		*N* = 12		*N* = 14		*N* = 8	
Palmitic acid	**0.522**	3.78	**0.647**	2.21	**0.723**	3.85	**0.798**	1.00	**0.840**	2.26
Stearic acid	**0.577**	1.84	**0.646**	2.26	**0.781**	2.58	0.164		0.082	
Oleic acid	**0.472**	10.4	0.166		0.390		0.360		**0.725**	2.26
Linoleic acid	**0.505**	5.62	0.149		0.425		−0.073		0.179	
DGLA	**0.560**	2.78	**0.496**	1.45	**0.743**	4.32	0.435		0.357	
Arachidonic acid	**0.394**	4.34	0.076		0.189		0.243		−0.088	
EPA	**0.391**	3.67	**0.487**	3.76	0.446		−0.213		0.372	
DHA	**0.425**	4.24	**0.420**	3.56	**0.625**	1.81	0.002		0.383	

*Note:* Pearson’s correlation between phosphatidylcholines and the 8 free fatty acids (palmitic acid, stearic acid, oleic acid, linoleic acid, DGLA, arachidonic acid, EPA, and DHA) in the nondiabetes group plus. DGLA, dihomo‐γ‐linolenic acid; NDM, patients without diabetes mellitus; DMH‐NDM, patients with Type 2 diabetes in remission after Roux‐en‐Y gastric bypass (RYGB) surgery; DMH‐DMH, patients with Type 2 diabetes not in remission after RYGB. The correlations written in bold had a significant (< 0.05) Pearson correlation with phosphatidylcholines and were therefore included in the stepwise multiple regression model.

Abbreviations: EPA, eicosapentaenoic acid; DHA, docosahexaenoic acid; P. Corr, Pearson’s correlation; VIF, variance inflation factor.

^∗^Variance inflation factor of the free fatty acids in the final linear regression model (after excluding the free fatty acids with insignificant Pearson’s correlation).

In line with the stronger correlations of FFA to PC compared to TAG, the adjusted *R* ^2^‐values in Table 8 indicate that compared to TAG, a greater part of the variance of PC could be explained by the FFA (Table 8). The linear regression of FFA on PC in the nondiabetes group had adjusted R^2^‐values at the respective timepoints ranging from 0.339 to 0.549, whereas they ranged from 0.491 to 0.688 (when excluding the value at 24 months because of multicollinearity) and 0.445 to 0.687 in the diabetes groups with and without remission, respectively (Table 8). Thus, at many timepoints, the FFA explained more than half of the variance in PC in the three groups.

**Table 8:** Stepwise linear regression for phosphatidylcholines and free fatty acids.

Phosphatidylcholines	Standardized slope coefficient	*p* value	Adjusted *R* ^2^	*F*‐change	*F*‐change *p* value
**NDM**					
Presurgery, *n* = 148					
**DGLA**	**0.491**	**2 × 10** ^**−**^ **^12^**	**0.446**	**119.5**	**1 × 10** ^**−**^ **^20^**
**Stearic acid**	**0.366**	**5 × 10** ^**−**^ **^8^**	**0.546**	**32.94**	**5 × 10** ^**−**^ **^8^**
EPA			0.547	2.625	0.107
3 months, *n* = 149					
**Palmitic acid**	**0.369**	**3 × 10** ^**−**^ **^6^**	**0.353**	**81.58**	**9 × 10** ^**−**^ **^16^**
**Stearic acid**	**0.286**	**1 × 10** ^**−**^ **^4^**	**0.407**	**14.52**	**2 × 10** ^**−**^ **^4^**
**DHA**	**0.227**	**0.001**	**0.450**	**12.31**	**0.001**
DGLA			0.457	2.928	0.089
6 months, *n* = 110					
**Palmitic acid**	**0.267**	**0.004**	**0.337**	**56.42**	**2 × 10** ^**−**^ **^11^**
**DGLA**	**0.226**	**0.011**	**0.429**	**18.33**	**4 × 10** ^**−**^ **^5^**
**DHA**	**0.245**	**0.001**	**0.477**	**10.92**	**0.001**
**Stearic acid**	**0.200**	**0.035**	**0.494**	**4.56**	**0.035**
Linoleic acid			0.506	3.451	0.066
12 months, *n* = 98					
**DGLA**	**0.362**	**2 × 10** ^**−**^ **^4^**	**0.260**	**35.14**	**5 × 10** ^**−**^ **^8^**
**Stearic acid**	**0.330**	**0.001**	**0.339**	**12.37**	**0.001**
DHA			0.350	2.605	0.110
24 months, *n* = 59					
**Stearic acid**	**0.429**	**2 × 10** ^**−**^ **^4^**	**0.441**	**46.75**	**6 × 10** ^**−**^ **^9^**
**Palmitic acid**	**0.415**	**3 × 10** ^**−**^ **^4^**	**0.549**	**14.61**	**3 × 10** ^**−**^ **^4^**
Arachidonic acid			0.554	1.637	0.206
**DMH-NDM**					
Presurgery, *n* = 33					
**DGLA**	**0.582**	**4 × 10** ^**−**^ **^5^**	**0.611**	**51.21**	**5 × 10** ^**−**^ **^8^**
**Stearic acid**	**0.356**	**0.006**	**0.688**	**8.63**	**0.006**
Palmitic acid			0.687	0.923	0.345
3 months, *n* = 33					
**DHA**	**0.470**	**0.002**	**0.425**	**24.7**	**2 × 10** ^**−**^ **^5^**
**Palmitic acid**	**0.411**	**0.005**	**0.545**	**9.18**	**0.005**
DGLA			0.552	1.43	0.242
6 months, *n* = 20					
**DGLA**	**0.566**	**0.002**	**0.470**	**17.9**	**0.001**
**Palmitic acid**	**0.420**	**0.013**	**0.614**	**7.73**	**0.013**
Oleic acid			0.638	2.14	0.163
12 months, *n* = 18					
**Arachidonic acid**	**0.722**	**0.001**	**0.491**	**17.4**	**0.001**
DGLA			0.556	3.35	0.087
24 months, *n* = 13					
**DHA**	**0.621**	**1 × 10** ^**−**^ **^4^**	**0.734**	**34.1**	**1 × 10** ^**−**^ **^4^**
**Linoleic acid**	**0.299**	**0.026**	**0.879**	**14.1**	**0.004**
**DGLA**	**0.250**	**0.045**	**0.916**	**5.43**	**0.045**
EPA			0.908	0.223	0.650
**DMH-DMH**					
Presurgery, *n* = 20					
**Stearic acid**	**0.506**	**0.010**	**0.295**	**8.96**	**0.008**
**Linoleic acid**	**0.419**	**0.027**	**0.445**	**5.85**	**0.027**
EPA			0.444	0.969	0.340
3 months, *n* = 20					
**Palmitic acid**	**0.698**	**5 × 10** ^**−**^ **^5^**	**0.386**	**13.0**	**0.002**
**EPA**	**0.552**	**0.001**	**0.687**	**18.3**	**0.001**
Stearic acid			0.669	0.062	0.806
6 months, *n* = 12					
**Stearic acid**	**0.781**	**0.003**	**0.571**	**15.6**	**0.003**
Palmitic acid			0.642	2.98	0.118
12 months, *n* = 14					
**Palmitic acid**	**0.798**	**0.001**	**0.607**	**21.1**	**0.001**
24 months, *n* = 10					
**Palmitic acid**	**0.84**	**0.009**	**0.656**	**14.4**	**0.009**
Oleic acid			0.618	0.398	0.556

*Note:* Stepwise linear regression with phosphatidylcholines as the dependent variable and the eight free fatty acids (palmitic acid, stearic acid, oleic acid, DGLA, arachidonic acid, EPA, and DHA) as the independent variables. DGLA, dihomo‐γ‐linolenic acid; NDM, patients without diabetes mellitus; DMH‐NDM, patients with Type 2 diabetes in remission after Roux‐en‐Y gastric bypass (RYGB) surgery; DMH‐DMH, patients with Type 2 diabetes not in remission after RYGB. The slope coefficient, standardized slope coefficient, *p* value, and variance inflation factor are from the final model including only the free fatty acids with significant contribution to the model. Adjusted *R* ^2^, *F*‐change, and *F*‐change *p* value reflect the stepwise addition of the respective free fatty acids in the model. The steps marked with bold are the FFA’s included in the model, while the steps not marked with bold are showing the adjusted *R*‐value and *F*‐change for the adding of the first FFA with an insignificant contribution to the model which is therefore not a part of the final model. The variance inflation factor was < 5 for all regressions.

Abbreviations: DHA, docosahexaenoic acid; EPA, eicosapentaenoic acid.

The omega‐6 fatty acids DGLA and arachidonic acid had the highest adjusted *R* ^2^‐values for PC in the remission group except at 3 and 24 months, where DHA was dominating (Table 8). In the group with persistent diabetes, the saturated palmitic acid and stearic acid were the dominating FFA driving the correlation to PC. Similar to the nondiabetes group, the fatty acids explained more of the variance in PC in the diabetes groups compared to the variance explained in TAG, amounting up to 2.7‐fold more.

## 5. Discussion

In this study, we investigated the relationship between eight FFA, TAG, and PC after RYGB to get insight into changes in lipid metabolism that might be causally related to postsurgery diabetes remission. To assess this hypothesis, we chose to investigate 3 groups of patients: a nondiabetes group, a group with Type 2 diabetes and postsurgery diabetes remission, and a group with Type 2 diabetes that persisted after RYGB. We have demonstrated an initial postsurgery decrease in the correlations between FFA and TAG and PC, respectively, followed by an increase after 6 months. At all timepoints, the FFA explained more of the variance in PC than in TAG, but diabetes status influenced the relationship between concentrations of FFA, TAG, and PC. The omega‐3 fatty acids were more strongly correlated to TAG in the group with diabetes remission than in the nondiabetes group. The low plasma concentration of omega‐6 fatty acid DGLA showed a surprisingly high correlation to both TAG and PC in all three groups. Unexpectedly, in the diabetes group without remission, a nonsignificant relation between stearic acid and oleic acid indicated a possible impaired conversion of stearic acid to oleic acid compared with the groups with remission, suggesting that regulatory metabolic pathways of lipid metabolism also might be implicated in post‐RYGB diabetes remission.

We found a significant correlation between PC and TAG at all timepoints in the nondiabetes group, at three timepoints in the diabetes group with remission, and only marginally at presurgery in the diabetes group without remission. This might suggest that the patients in the diabetes groups represent a continuum of pathophysiological changes in relation to the interaction of beta‐cell function and insulin resistance resulting in differences in glucose and lipid metabolism.

We found a tendency for the correlations between PC and TAG, and the adjusted R‐values of the linear regression for the FFA and TAG to decrease after surgery, followed by an increase after 6 months, especially in the nondiabetes group. This may suggest that in all three groups, the postsurgery decrease that was followed by an increase after 3 months for PC [6], TAG (Table S1), and most of the FFA [7] does not merely reflect a decreased intake and absorption, but also an altered lipid metabolism.

However, as we did not find this pattern in the correlations in the group without remission, this could indicate that the regulatory mechanisms causing the changes in the correlations are impaired in this group after surgery. We speculate whether this could potentially be part of the physiological difference underlying why some diabetes patients obtain remission after RYGB and some do not.

DGLA, palmitic acid, stearic acid, and oleic acid were the four fatty acids with the highest Pearson correlations to TAG and PC presurgery in the nondiabetes group. As palmitic acid, stearic acid, and oleic acid were among the most abundant fatty acids, it makes sense that they would have a higher correlation to TAG and PC than some of the less abundant FFA. However, in the nondiabetes group, DGLA had the highest correlation of all eight fatty acids to both TAG and PC presurgery. Given that the concentration of DGLA was almost 20 times lower than the most abundant fatty acid, palmitic acid, it is interesting that it explained most of the R‐value of the FFA correlation to both TAG and PC before RYGB surgery and again after 12 months postsurgery in the nondiabetes group. This suggests that the strong correlation might be caused by regulatory mechanisms rather than merely incorporation of DGLA into TAG and PC.

A study showed that DGLA inhibits uptake of LDL in foam cells [22]. It has been suggested that inflammation plays a role in the development of dyslipidemia [23] and PGE1 is a highly anti‐inflammatory metabolite downstream from DGLA [24]. Thus, there are some indications that DGLA could potentially affect levels of TAG and PC.

Besides being incorporated into TAG for energy storage, into phospholipids as the main part of the phospholipid bilayer of cell membranes, and serving as precursors to various signaling molecules, such as prostaglandins and steroids, FFA can specifically affect changes in lipid metabolism by altering gene expression patterns [25]. This effect has only been reported for polyunsaturated fatty acids and not for monounsaturated or saturated fatty acids which serve as the main energy source among long‐chain fatty acids. It has therefore been suggested that this polyunsaturated‐specific effect might be most influential on the generation of phospholipids rather than energy metabolism regulation in general [26]. We found a higher correlation between the FFA and PC compared to TAG which supports this assumption. Another possible regulatory mechanism of PC and TAG synthesis could be through the activation of CTP:phosphocholine cytidylyltransferase which regulates the flux of DAG, a substrate for both TAG and PC synthesis [14], that facilitates the rate‐limiting step in condensation of DAG with choline to form PC, and is activated by fatty acids [27]. To our knowledge, no studies have investigated regulatory mechanisms in lipid metabolism for DGLA specifically.

The correlation of EPA and DHA to TAG was higher in the diabetes group with remission compared to the nondiabetes group at most timepoints, which could suggest a mechanism by which EPA and DHA and their anti‐inflammatory metabolites assist in restoration of normal glucose metabolism. A meta‐analysis including 13 studies with a total of 36,542 included individuals has indicated with a risk ratio of 0.74 (0.62–0.89) that higher omega‐3 fatty acid intake is associated with a reduction in risk of developing Type 2 diabetes [28]. In our previous study, no difference in EPA and DHA plasma levels was found between the three groups at any timepoint except between the nondiabetes group and the diabetes group with remission presurgery [7]. Thus, a potential beneficial effect of EPA and DHA on glucose metabolism would, in our cohort, not be reflected in the plasma levels but might rather suggest a difference in the action of how EPA and DHA affect the synthesis and metabolism of TAG and PC that resulted in a stronger correlation of EPA and DHA to TAG in the diabetes group with remission.

The fact that we found a slope coefficient for the regression with stearic acid and oleic acid to be nonsignificant in the group with persistent diabetes could indicate that the subjects in this group did not convert stearic acid to oleic acid as efficiently as the other two groups. However, the oleic acid plasma concentration was not significantly different between groups. Thus, in the group with persistent diabetes, more oleic acid might be produced from the breakdown of TAG compared to the production from stearic acid by the enzyme delta‐9‐desaturase [29].

Studies suggest that while palmitic acid contributes to the development of insulin resistance, oleic acid benefits the cells and lessens the weakening of the insulin signaling pathway caused by palmitic acid [30, 31]. Thus, the possible impaired conversion of stearic acid to oleic acid might be a contributing factor to why some diabetes patients do not obtain remission after RYGB suggesting delta‐9‐desaturase to be a possible target in treating Type 2 diabetes [29].

### 5.1. Methodological Considerations, Limitations, and Future Directions

This study has several strengths, including a well‐characterized cohort, long‐term follow‐up, and detailed quantification of selected FFA and complex lipids. Nonetheless, certain limitations should be acknowledged. The observational design precludes causal inference, and residual confounding from unmeasured factors, such as dietary intake, cannot be excluded. Furthermore, differences in preoperative insulin treatment between diabetes subgroups could reflect disease severity and may act as a potential confounder, although no participants received insulin postsurgery. To get an even better overview of the FFA metabolism, it would have been beneficial to measure metabolites from all relevant pathways; however, this was not possible with our method. Furthermore, it would have strengthened the study to have detailed dietary data from the participants.

From an analytical perspective, the GC‐MS method applied relied on separation of nonesterified from esterified fatty acids before derivatization. While the extraction procedure is well‐established, minor contamination of the FFA fraction from phospholipids cannot be entirely excluded. Given these potential challenges, future methodological developments should aim to reduce uncertainty in FFA quantification, for example, by employing direct analysis approaches, such as LC‐MS or by validating results across multiple extraction protocols.

Future research should focus on further elucidating the role of delta‐9‐desaturase activity in the conversion of stearic acid to oleic acid and its possible impact on diabetes remission after RYGB. In addition, the strong associations between DGLA and both PC and TAG warrant more detailed mechanistic investigation.

## 6. Conclusion

The eight measured FFA explained markedly more of the PC variation compared to the variation in TAG, indicating that FFA might be involved to a higher extent in the regulation of synthesis and metabolism of PC than of TAG.

It is proposed that the postsurgery decrease in the correlations between FFA and TAG and PC, respectively, that was followed by an increase after 6 months indicates that not only ingestion and absorption of lipids are altered after RYGB, but that regulatory mechanisms of lipid metabolism also might be altered.

As this pattern in correlations was not seen in the group without diabetes remission and followed a similar decrease and increase in FFA, TAG, and PC in all three groups, it is speculated whether this reflects part of the physiological differences explaining why some patients obtain diabetes remission after RYGB and others do not.

The fact that DGLA, which is one of the least abundant FFA, was the FFA with the highest correlation to TAG and PC both presurgery and again after 12 months in all three groups suggests that the correlation might be caused by a regulatory mechanism rather than by integration of DGLA into TAG and PC.

The unexpected nonsignificant slope coefficient for stearic acid, which was explaining the variation in oleic acid in the group without remission, indicates a possible inefficient conversion of stearic acid to oleic acid with the enzyme delta‐9‐desaturase.

Study limitations include the restricted lipidomic scope, lack of dietary data, and potential analytical uncertainty in FFA extraction, as discussed above; future studies should address these issues and investigate key mechanistic links, such as delta‐9‐desaturase activity and the role of DGLA in lipid metabolism after RYGB.

**Nomenclature**
DAG: Diacylglycerols
DGLA: Dihomo‐γ‐linolenic acid
DHA: Docosahexaenoic acid
DMH‐DMH: Patients with diabetes mellitus not in remission after RYGB
DMH‐NDM: Patients with diabetes mellitus in remission after RYGB
EPA: Eicosapentaenoic acid
FFA: Free fatty acids
HbA1c: Glycated hemoglobin
HDL: High‐density lipoprotein
HOMA2‐IR: Homeostatic Model Assessment for Insulin Resistance
LDL: Low‐density lipoprotein
NDM: Patients without diabetes mellitus
PC: Phosphatidylcholines
RYGB: Roux‐en‐Y gastric bypass
TAG: Triglycerides
VLDL: Very‐low‐density lipoprotein cholesterol

## Ethics Statement

This study was performed in accordance with the Helsinki Declaration and was approved by the Scientific Ethics Committee of the Capital Region, Denmark, protocols number HD2009–78 and H‐6‐2014‐029, and by the Danish Data Protection Agency.

## Consent

All participants in the study gave their informed consent in writing.

## Disclosure

This research was performed as part of the employment of the authors’ following three employers:

1. Department of Clinical Biochemistry, Nordsjællands Hospital, University of Copenhagen, Hillerød, Denmark

2. Department of Clinical Biochemistry, Copenhagen University Hospital Hvidovre, Hvidovre, Denmark

3. Department of Endocrinology, Copenhagen University Hospital Hvidovre, Hvidovre, Denmark

## Conflicts of Interest

The authors declare no conflicts of interest.

## Author Contributions

Freja Eriksen performed data collection, analyzed and researched data, and wrote the manuscript. Sten Madsbad designed the study, contributed to discussion, and reviewed/edited the manuscript. Mogens Fenger designed the study, contributed to discussion, and reviewed/edited the manuscript. Elin R. Carlsson performed data collection and contributed to the discussion and reviewed/edited the manuscript. All authors take responsibility for the contents of this article.

## Funding

The research did not receive any specific funding.

## Supporting Information

The supporting information contains one table (Table S1) which report data as mean concentrations (95% CI) of the 8 FFA, triglyceride, phosphatidylcholine, ApoA1 and ApoB in the three diabetes groups at the time points: presurgery, 3, 6, 12 and 24 months postsurgery.

## Supporting information


**Supporting Information** Additional supporting information can be found online in the Supporting Information section.

## Data Availability

The datasets used and/or analyzed during this study are available from the corresponding author upon reasonable request.
