# Supplementary material for: Free Fatty Acids and Complex Lipids in Patients With Severe Obesity Treated With Roux‐En‐Y Gastric Bypass: Impact of Diabetes Status
Source: Biochem Res Int. 2025 Dec 8;2025:1805140. doi: 10.1155/bri/1805140 (PMC12707549; doi:10.1155/bri/1805140)
Supplement: Supplementary file 1 — Supporting Information Additional supporting information can be found online in the Supporting Information section. [file BRI-2025-1805140-s001.docx]

Supplemental material

| mmol/L | Pre-surgery  Mean (95% CI) | 3 months Post-surgery Mean (95% CI) | 6 months Post-surgery Mean (95% CI) | 12 months Post-surgery Mean (95% CI) | 24 months Post-surgery  Mean (95% CI) |
| --- | --- | --- | --- | --- | --- |
| **NDM** | N=149 | N=149 | N=111 | N=100 | N=65 |
| Palmitic acid | 1.736 (1.662-1.810) | 1.500 (1.443-1.558) | 1.462 (1.402-1.523) | 1.497 (1.4301-1.564) | 1.519 (1.424-1.615) |
| Stearic acid | 0.531 (0.511-0.551) | 0.423 (0.409-0.438) | 0.459 (0.442-0.477) | 0.488 (0.469-0.506) | 0.497 (0.472-0.523) |
| Oleic acid | 0.855 (0.819-0.892) | 0.859 (0.826-0.892) | 0.850 (0.809-0.891) | 0.867 (0.828-0.905) | 0.837 (0.786-0.888) |
| Linoleic acid | 0.872 (0.832-0.912) | 0.711 (0.676-0.746) | 0.752 (0.709-0.794) | 0.799 (0.7535-0.845) | 0.820 (0.767-0.874) |
| DGLA | 0.0983 (0.0919-0.105) | 0.0634 (0.0595-0.0673) | 0.0758 (0.0697-0.0819) | 0.0838 (0.0777-0.0900) | 0.0973 (0.0858-0.109) |
| Arachidonic acid | 0.462 (0.435-0.488) | 0.446 (0.424-0.468) | 0.442 (0.417-0.466) | 0.435 (0.410-0.460) | 0.426 (0.392-0.459) |
| EPA | 0.0393 (0.0357-0.0428) | 0.0262 (0.0239-0.0284) | 0.0301 (0.0270-0.0333) | 0.0332 (0.0300-0.0365) | 0.0399 (0.0344-0.0455) |
| DHA | 0.122 (0.115-0.130) | 0.117 (0.110-0.123) | 0.119 (0.0502-0.127) | 0.119 (0.111-0.127) | 0.120 (0.109-0.131) |
| Triglyceride | 1.511 (1.390-1.632)  N=145 | 1.085 (1.0278-1.142) | 1.009 (0.952-1.065)  N=131 | 0.961 (0.903-1.018)  N=134 | 0.974 (0.901-1.047)  N=128 |
| PC | 1.924 (1.860-1.985) | 1.625 (1.585-2.158) | 1.723 (1.674-1.772) | 1.823 (1.765-1.882)  N=98 | 1.928 (1.841-2.015)  N=60 |
| **DMH-NDM** | N=33 | N=33 | N=21 | N=18 | N=13 |
| Palmitic acid | 1.816 (1.622-2.010) | 1.569 (1.432-1.706) | 1.606 (1.393-1.818) | 1.439 (1.290-1.588) | 1.795 (1.439-2.150) |
| Stearic acid | 0.528 (0.482-0.573) | 0.452 (0.413-0.492) | 0.466 (0.415-0.517) | 0.472 (0.431-0.513) | 0.599 (0.499-0.699) |
| Oleic acid | 0.933 (0.847-1.020) | 0.936 (0.851-1.021) | 0.930 (0.830-1.030) | 0.906 (0.769-1.043) | 0.987 (0.742-1.231) |
| Linoleic acid | 0.870 (0.753-0.986) | 0.707 (0.629-0.784) | 0.747 (0.634-0.859) | 0.780 (0.669-0.890) | 0.869 (0.698-1.041) |
| DGLA | 0.0975 (0.0799-0.115) | 0.0658 (0.0562-0.0754) | 0.0796 (0.0673-0.0919) | 0.0772 (0.0648-0.0897) | 0.105 (0.0846-0.126) |
| Arachidonic acid | 0.510 (0.439-0.581) | 0.503 (0.430-0.577) | 0.460 (0.394-0.525) | 0.486 (0.384-0.587) | 0.512 (0.366-0.657) |
| EPA | 0.0532 (0.0426-0.0638) | 0.0310 (0.0260-0.0360) | 0.0285 (0.0227-0.0342) | 0.0305 (0.0251-0.0359) | 0.0563 (0.037-0.0754) |
| DHA | 0.140 (0.120-0.160) | 0.125 (0.109-0.141) | 0.119 (0.101-0.137) | 0.119 (0.101-0.138) | 0.150 (0.102-0.197) |
| Triglyceride | 2.148 (1.452-2.844) | 1.275 (1.051-1.500) | 1.111 (0.966-1.256)  N=30 | 1.0642 (0.856-1.272)  N=26 | 1.190 (0.904-1.475)  N=25 |
| PC | 1.876 (1.687-2.065) | 1.624 (1.521-1.728) | 1.730 (1.604-1.855)  N=20 | 1.733 (1.616-1.850) | 2.114 (1.769-2.459) |
| **DMH-DMH** | N=20 | N=20 | N=13 | N=15 | N=10 |
| Palmitic acid | 1.597 (1.380-1.814) | 1.328 (1.175-1.481) | 1.344 (1.158-1.530) | 1.340 (1.156 -1.523) | 1.315 (1.118-1.512) |
| Stearic acid | 0.496 (0.448-0.545) | 0.394 (0.359-0.430) | 0.428 (0.365-0.491) | 0.448 (0.397-0.500) | 0.444 (0.393-0.494) |
| Oleic acid | 0.809 (0.661-0.957) | 0.773 (0.697-0.848) | 0.787 (0.709-0.865) | 0.736 (0.666-0.807) | 0.757 (0.628-0.885) |
| Linoleic acid | 0.692 (0.577-0.807) | 0.554 (0.493-0.615) | 0.612 (0.526-0.697) | 0.647 (0.549-0.746) | 0.711 (0.634-0.788) |
| DGLA | 0.0837 (0.0709-0.0966) | 0.0558 (0.0485-0.0631) | 0.0689 (0.0540-0.0838) | 0.0708 (0.0572-0.0844) | 0.0707 (0.0579-0.0836) |
| Arachidonic acid | 0.454 (0.374-0.533) | 0.420 (0.361-0.478) | 0.444 (0.361-0.528) | 0.387 (0.324-0.450) | 0.425 (0.354-0.496) |
| EPA | 0.0481 (0.0373-0.0591) | 0.0290 (0.0168-0.0412) | 0.0347 (0.0224-0.0471) | 0.0419 (0.0266-0.0573) | 0.0519 (0.0243-0.0795) |
| DHA | 0.122 (0.0952-0.148) | 0.108 (0.0807-0.135) | 0.117 (0.083-0.152)  N=12 | 0.104 (0.0749-0.134) | 0.127 (0.0849-0.170) |
| Triglyceride | 2.114 (1.537-2.690) | 1.193 (0.997-1.388) | 1.193 (0.988-1.397)  N=20 | 1.0947 (0.868-1.322)  N=19 | 1.0563 (0.816-1.297)  N=19 |
| PC | 1.860 (1.653-2.065) | 1.555 (1.396-1.715) | 1.661 (1.422-1.901) | 1.732 (1.575-1.888) | 1.746 (1.543-1.949)  N=8 |

**Table S1:** Data are reported as mean concentrations (95% CI) of the 8 FFA, triglyceride, phosphatidylcholine, ApoA1 and ApoB in the three diabetes groups at the time-points: pre-surgery, 3, 6, 12 and 24 months post-surgery. DGLA, Dihomo-γ-linolenic acid; EPA, Eicosapentaenoic acid; DHA, Docosahexaenoic acid; PC, phosphatidylcholine; NDM, Patients without diabetes mellitus; DMH-NDM, Patients with DM in remission after Roux-en-Y gastric bypass surgery (RYGB); DMH-DMH, patients with DM not in remission after RYGB.
